# Predicting Infectious Disease Incidence After Flooding Using Artificial Intelligence Models: A Retrospective Pre–Post Cohort Study Using Routinely Collected EHR Data

**DOI:** 10.1002/hsr2.71470

**Published:** 2025-11-06

**Authors:** Mehdi Safari, Alireza Zali, Hossein Hatami, Elnaz Amanzadeh Jajin, Meisam Akhlaghdoust

**Affiliations:** ^1^ Department of Public Health, School of Public Health & Environmental and Occupational Hazards Control Research Center Shahid Beheshti University of Medical Sciences Tehran Iran; ^2^ Shahid Beheshti University of Medical Sciences Tehran Iran; ^3^ Functional Neurosurgery Research Center, Shohada Tajrish Comprehensive Neurosurgical Center of Excellence Shahid Beheshti University of Medical Sciences Tehran Iran; ^4^ USERN Office, Functional Neurosurgery Research Center Shahid Beheshti University of Medical Sciences Tehran Iran

**Keywords:** artificial intelligence, floods, incidence, infectious diseases, machine learning

## Abstract

**Background and Aims:**

Natural disasters, particularly floods, significantly increase infectious disease risk through environmental contamination and healthcare system disruption. Despite well‐documented flood‐disease associations, predictive models for post‐disaster epidemiological surveillance remain limited. We aimed to develop and validate machine learning algorithms to predict infectious disease incidence following flood events.

**Methods:**

We conducted a retrospective pre–post cohort study using routinely collected electronic health records from Firuzkuh County health centers, comparing a 30‐day pre‐flood cohort (July–August 2021; *n* = 461) with a 30‐day post‐flood cohort (July–August 2022; *n* = 478). Five classifiers (Random Forest, Logistic Regression, linear SVM, Gradient Boosting, and ANN) were trained and evaluated on a held‐out test set using AUC.

**Results:**

Post‐flood infectious disease prevalence increased significantly from 39.5% to 47.3% (*p* < 0.001), with an odds ratio of 1.38 (95% CI: 1.09–1.75) and attributable risk of 7.8 percentage points. Among machine learning models, Random Forest achieved the highest predictive performance (AUC = 0.76), followed by Gradient Boosting (0.74), Artificial Neural Network (0.72), Support Vector Machine (0.71), and Logistic Regression (0.69). Age and visit date emerged as the most important predictive features across all models. Unexpectedly, younger patients (mean age 51.0 years) showed higher post‐flood infectious disease rates compared to older patients (mean age 57.9 years) in the pre‐flood period.

**Conclusion:**

Machine learning models demonstrated moderate predictive performance for post‐flood infectious disease occurrence. While results show feasibility for AI‐based disaster epidemiology, the modest performance indicates that incorporating additional environmental and socioeconomic variables is essential for developing clinically actionable prediction systems for public health emergency response.

## 1 Introduction

Natural disasters inflict profound disruptions on human health systems, with floods representing the most widespread and destructive among them. Over the past decade, flooding events have claimed approximately 53,000 lives while affecting more than two billion people worldwide [1, 2, 3]. According to the International Disaster Database (EM‐DAT), floods have caused more devastating consequences than any other natural calamity in the 21st century, both in terms of human impact and economic devastation [4, 5]. The health consequences of flooding extend far beyond immediate physical trauma. Floodwaters create conditions conducive to infectious disease transmission through multiple interconnected pathways: environmental contamination disrupts sanitation infrastructure, population displacement leads to overcrowded temporary shelters with inadequate hygiene facilities, and damaged healthcare systems struggle to maintain routine preventive services [3]. These cascading effects consistently precipitate infectious disease outbreaks, with well‐documented increases in diarrheal diseases, cholera, dengue fever, malaria, and acute respiratory infections following major flood events [1, 2, 3, 6, 7, 8]. Iran occupies a position of particular vulnerability in the global disaster landscape, ranking among the ten most disaster‐prone nations with exposure to more than 30 of the 40 recognized natural disaster types [1, 9, 10]. This geographic and climatic predisposition, combined with rapid urbanization and climate change, necessitates sophisticated approaches to disaster preparedness and response planning.

The convergence of artificial intelligence and public health offers unprecedented opportunities to transform disaster epidemiology from reactive to predictive science. Recent systematic reviews have demonstrated the effectiveness of machine learning approaches in infectious disease surveillance and outbreak prediction [4, 6, 7, 11]. The complexity of disaster‐health interactions—involving environmental, social, and biological factors—makes them particularly suitable for machine learning approaches capable of identifying nonlinear patterns and interactions that traditional statistical methods might overlook. Despite growing recognition of artificial intelligence's potential in disaster response, few studies have specifically addressed post‐flood infectious disease prediction using machine learning methodologies. This gap represents a critical missed opportunity for public health preparedness, particularly given the increasing frequency and intensity of extreme weather events associated with climate change. To address this knowledge gap, we developed and validated multiple machine learning algorithms for predicting infectious disease incidence following flood disasters. Using data from a major flood event in Firuzkuh County, Iran, we sought to identify key predictive factors and evaluate the feasibility of AI‐driven approaches for disaster epidemiological surveillance.

## 2 Materials and Methods

### 2.1 Study Design and Geographic Setting

We conducted a retrospective pre–post cohort study with temporal comparison, leveraging individual‐level EHR data to examine infectious disease patterns before and after a major flooding event. The study was conducted in Firuzkuh County, located in Tehran Province, Iran (35°45′25″ N, 52°46′13″ E). This mountainous region, with a population of 33,558 residents across 11,700 households, experienced catastrophic flooding on July 29, 2022. The disaster resulted in 15 fatalities, 16 missing persons, 12 serious injuries, and extensive agricultural damage affecting over 350 hectares of cultivated land.

### 2.2 Study Populations and Temporal Framework

Our analysis encompassed two distinct patient cohorts seeking care at Firuzkuh County health centers:

▪ Pre‐flood cohort (PF): Patients receiving care during the 30‐day period of July–August 2021 (*n* = 461).
▪ Post‐flood cohort (AF): Patients receiving care during the corresponding 30‐day period following the 2022 flood (July–August 2022, *n* = 478).

This temporal comparison design was specifically chosen to account for seasonal epidemiological patterns while isolating the flood's impact on infectious disease prevalence. The 30‐day observation window captures the critical period when most flood‐related infectious diseases manifest, based on established incubation periods and transmission dynamics documented in disaster epidemiology literature [3, 6, 8].

### 2.3 Data Collection and Variable Definition

Patient data were extracted from electronic health records maintained by Firuzkuh County health centers, which serve as the primary healthcare access points for the region's population. Our data collection protocol focused on four key predictor variables selected based on their clinical relevance and availability in routine health records:

1. Age: Recorded in years at time of healthcare encounter
2. Gender: Categorized as male or female
3. Underlying diseases: Binary variable indicating presence or absence of documented chronic conditions
4. Date of health center reference: Temporal variable capturing healthcare utilization timing

The primary outcome was diagnosis of an infectious or parasitic disease coded according to International Classification of Diseases, 10th Revision (ICD‐10) criteria by qualified medical personnel. Non‐communicable conditions were excluded.

### 2.4 Ethical Considerations

This study received approval from the Research Ethics Committees of the School of Public Health & Neuroscience Research Center, Shahid Beheshti University of Medical Sciences (approval number: IR.SBMU.PHNS.REC.1402.039). All participants provided informed written consent for inclusion in medical research and publication of anonymized data.

### 2.5 Machine Learning Methodology

i. Algorithm Selection and Rationale

We selected five complementary machine learning algorithms representing different methodological paradigms commonly employed in epidemiological prediction:

1. Logistic Regression (LR) served as our baseline linear model, chosen for its interpretability and widespread acceptance in medical research. The logistic function estimates infectious disease probability as:
$$P(y=1|X)=1/(1+e^{\Lambda }(-(\beta_{0}+\beta_{1}X_{1}+\beta_{2}X_{2}+\text{…}+\beta_{n}X_{n})))$$
  where *β* coefficients represent the log‐odds change associated with each predictor variable.
2. Random Forest (RF) was selected as a robust ensemble method capable of capturing nonlinear relationships and feature interactions without overfitting. The algorithm combines multiple decision trees using bootstrap aggregation:
$$\hat{y}=\left(1/B\right)\;\times \;𝚺(b=1\;to\;B)T_{\beta }\left(x\right)$$
  where *B* represents the number of trees and T_β_(x) denotes predictions from individual trees.
3. Support Vector Machine (SVM) was included for its effectiveness in binary classification tasks and superior generalization performance with limited sample sizes. The classification function is defined as:
$$f\left(x\right)\;=\;sign(𝚺(i=1\;to\;n)\alpha_{i}y_{i}K\left(x_{i},x\right)+b)$$
  where *α*_i_ are Lagrange multipliers, *y*_i_ represent class labels, and *K*(*x*_i_, *x*) is the kernel function.
4. Gradient Boosting (GB) was chosen as an iterative ensemble method that sequentially improves prediction accuracy by combining weak learners, offering superior performance for complex pattern recognition tasks.
5. Artificial Neural Network (ANN) provided a deep learning perspective, capable of capturing intricate nonlinear patterns and interactions that traditional methods might miss.

ii. Model Development and Validation
  Data preprocessing included standardization of continuous variables and encoding of categorical features. The complete data set was randomly partitioned into training (75%) and testing (25%) subsets, ensuring representative distribution of outcome variables in both partitions.
  Hyperparameter optimization was performed using Bayesian optimization techniques to maximize model performance while preventing overfitting. Model validation employed stratified cross‐validation during training, with final performance assessment conducted on the independent test set.
iii. Performance Evaluation
  Model discrimination was evaluated using the Area Under the Receiver Operating Characteristic Curve (AUC):
$$\mathrm{AUC}=\int 0¹\;\mathrm{TPR}\left({\mathrm{FPR}}^{-¹}\left(t\right)\right)\mathrm{dt}$$
  where TPR represents True Positive Rate and FPR represents False Positive Rate. AUC values range from 0.5 (random performance) to 1.0 (perfect discrimination). Feature importance was summarized as mean decrease in impurity for Random Forest and as absolute standardized coefficients for Logistic Regression and linear SVM. Permutation importance for GB/ANN was not reported due to instability with the small feature set.

### 2.6 Statistical Analysis

Comparative analysis between pre‐flood and post‐flood cohorts employed *χ* ^2^ tests for categorical variables and unpaired *t*‐tests for continuous variables. Effect size calculations included odds ratios, relative risks, and attributable risk estimates with 95% confidence intervals. Correlation analysis utilized Pearson correlation coefficients to examine relationships between predictor variables. All analyses were conducted using Python scientific computing libraries (NumPy, Pandas, Scikit‐learn and Matplotlib). Statistical significance was defined as *p* < 0.05, and all tests were two‐tailed. We distinguished pre‐specified primary analysis (flood impact on infectious disease prevalence) from exploratory analyses (correlation between variables and feature importance assessment). References for statistical methods are to standard works where possible, following guidelines for reporting statistics in the clinical research [12].

## 3 Results

### 3.1 Infectious Disease Prevalence Before and After Flooding

**Figure 1:**
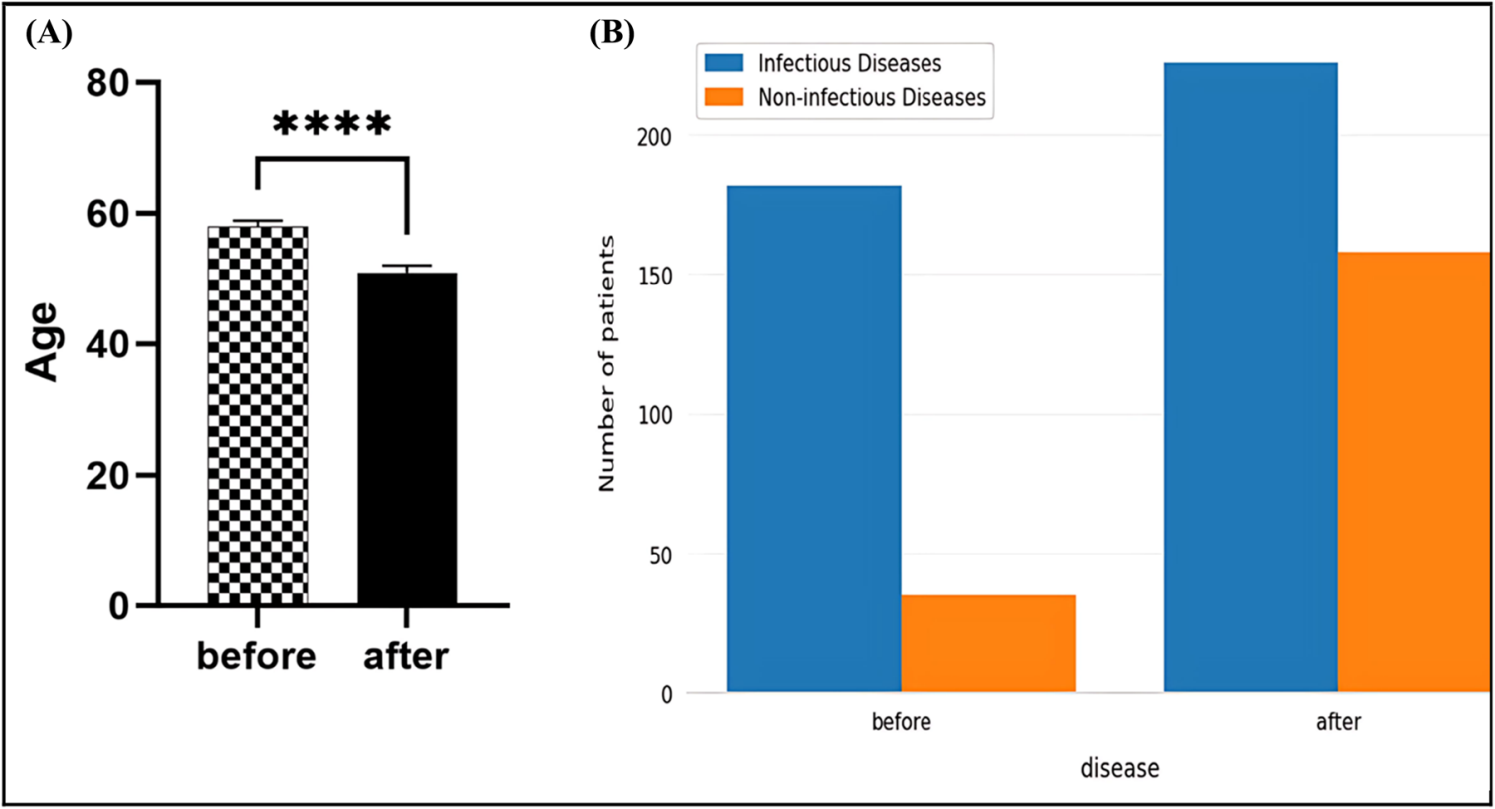
Demographics and infectious‐disease diagnoses before vs. after the July 2022 flood. (A) Infectious‐disease proportions were 182/461 (39.5%) pre‐flood and 226/478 (47.3%) post‐flood; *χ*² [1] = 5.81, *p* = 0.016. (B) Age distribution comparison between study periods Box plots display medians and interquartile ranges; whiskers denote 1.5×IQR; points indicate outliers.

Among the 939 patients who sought care at Firuzkuh health centers, 461 were treated during the pre‐flood period and 478 during the post‐flood period. The number of infectious disease cases was 182 in the pre‐flood group and 226 in the post‐flood group, representing 44.61% and 55.39% of total infectious disease cases, respectively (Figure 1A). When examining prevalence within each time period, infectious diseases affected 182 of 461 patients (39.5%) in the pre‐flood group compared to 226 of 478 patients (47.3%) in the post‐flood group. *χ*² analysis revealed a statistically significant association between flood occurrence and infectious disease prevalence ( *χ*² = 6.12, df = 1, *p* < 0.001) (Figure 1B). Effect size calculations demonstrated clinically meaningful associations: patients in the post‐flood period had 1.38 times higher odds of developing infectious diseases (OR = 1.38, 95% CI: 1.09–1.75). The relative risk showed infectious disease risk was 1.20 times higher in the post‐flood group (RR = 1.20, 95% CI: 1.05–1.37). The attributable risk in the exposed population was 7.8% points, indicating that 7.8% of infectious disease cases in the post‐flood period could be attributed to flood‐related factors. The number of exposed individuals per attributable case was 13, meaning that for every 13 individuals exposed to post‐flood conditions, one additional case of infectious disease could be attributed to flood‐related factors.

### 3.2 Age and Gender Distribution Patterns

Age analysis revealed an unexpected demographic pattern. An unpaired *t*‐test comparing age distributions between periods showed that patients in the pre‐flood group were significantly older (mean age ± SD = 57.94 ± 20.25 years) compared to patients in the post‐flood group (mean age ± SD = 50.99 ± 22.30 years) (*t* = 4.12, df = 937, *p* < 0.001, unpaired *t*‐test) (Figure 1B). This difference was statistically significant, indicating that younger patients were more likely to present with infectious diseases following the flood event.

Gender distribution analysis showed no significant differences between male and female patient numbers in the pre‐flood and post‐flood groups (*χ*² = 0.24, df = 1, *p* = 0.62), suggesting that gender was not a significant factor in post‐flood infectious disease patterns in this population.

### 3.3 Machine Learning Model Performance

**Figure 2:**
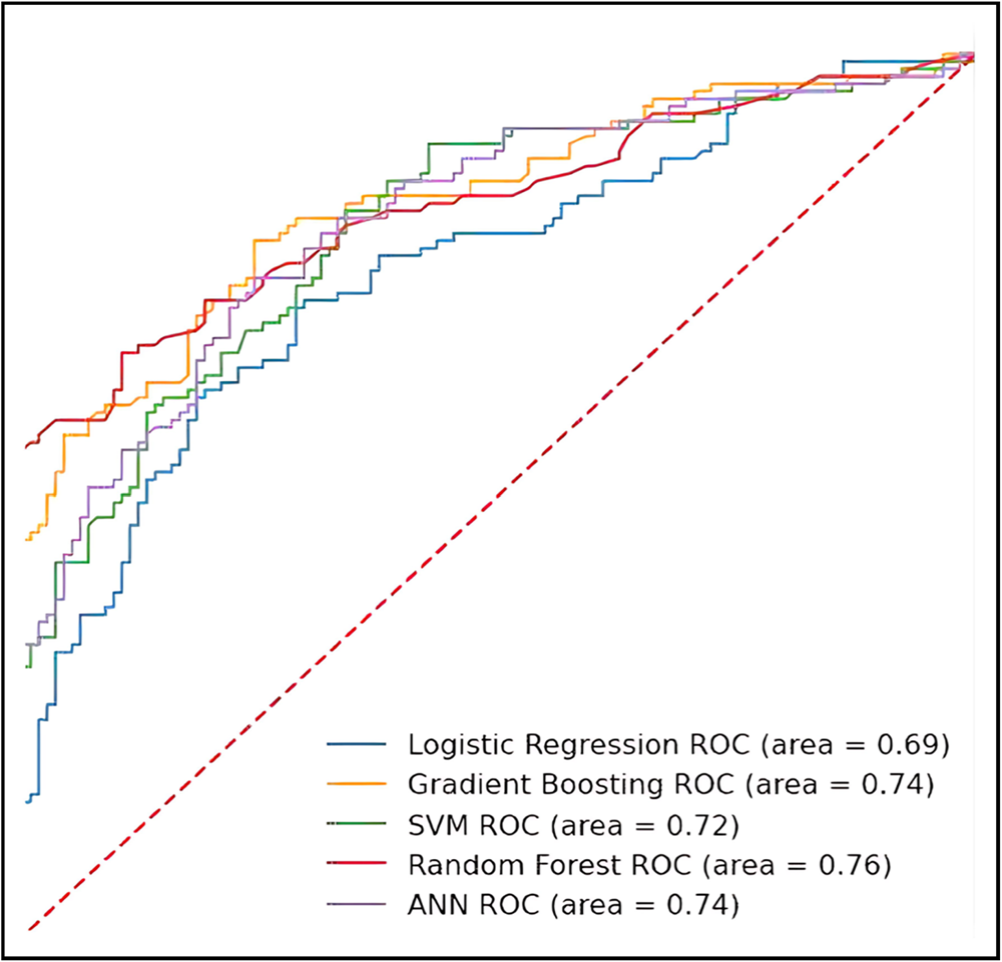
Receiver operating characteristic (ROC) curve analysis and area under the curve (AUC) performance comparison. ROC curves for all five machine learning algorithms with corresponding AUC values: Random Forest (0.76), Gradient Boosting (0.74), Artificial Neural Network (0.72), Support Vector Machine (0.71), and Logistic Regression (0.69). The diagonal reference line represents random performance (AUC = 0.50). Random Forest demonstrated superior discrimination capability, while all models achieved moderate predictive performance above chance levels.

Comparative evaluation of the five machine learning algorithms revealed varying predictive performance levels (Figure 2). Across models, test‐set AUC ranged from 0.69 to 0.76; Random Forest performed best, followed by Gradient Boosting and ANN, with Logistic Regression and linear SVM lower. The complete performance ranking showed moderate discrimination across all algorithms, with AUC values ranging from 0.69 to 0.76.

The superior performance of the Random Forest algorithm suggests that ensemble methods may be better suited for capturing the complex relationships inherent in post‐disaster infectious disease patterns compared to linear approaches.

### 3.4 Feature Importance Analysis

**Figure 3:**
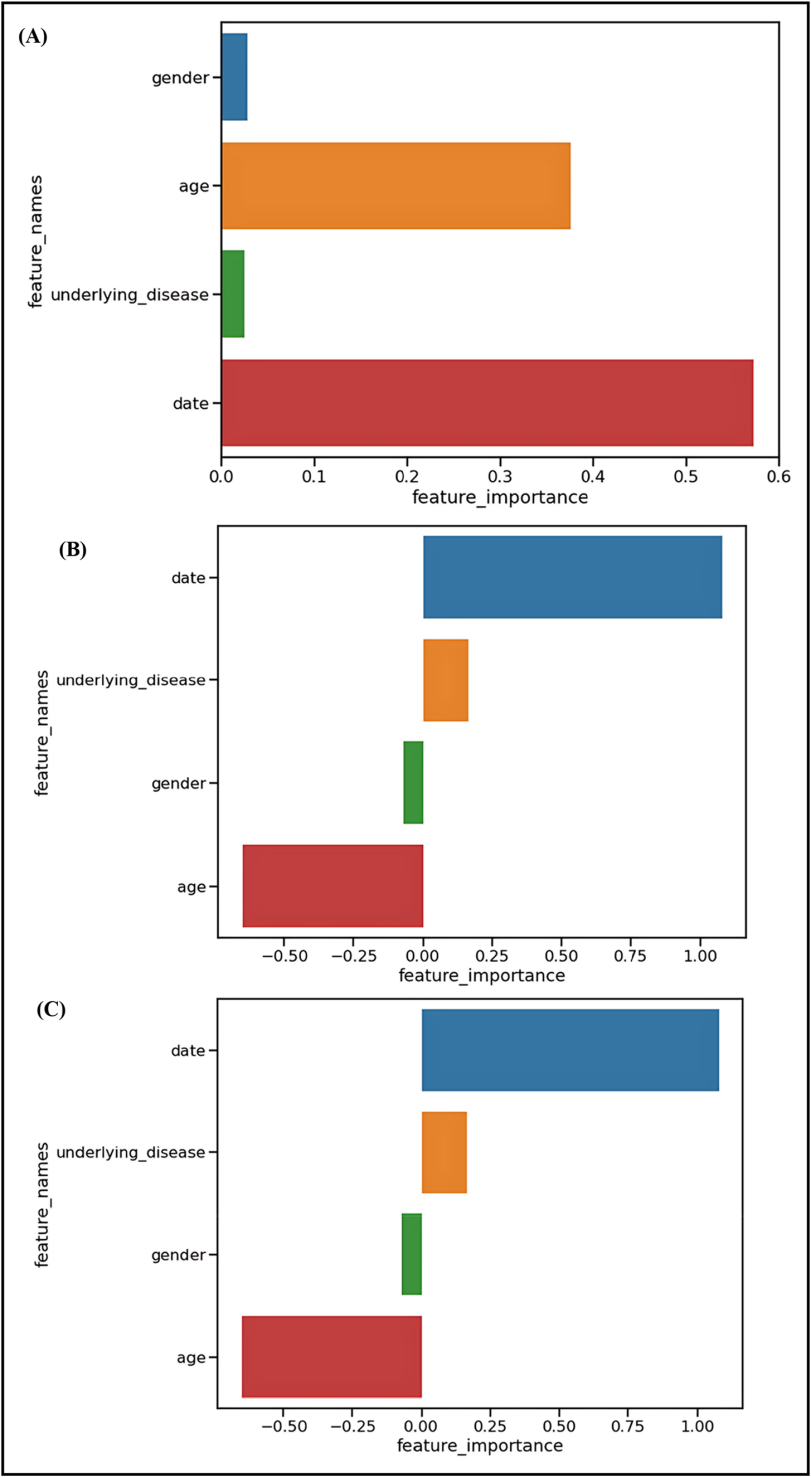
Feature importance rankings by machine learning algorithm. (A) Random Forest feature importance based on Gini impurity reduction, showing visit date (0.572) and age (0.376) as dominant predictors. (B) Logistic Regression coefficient magnitudes, with positive values indicating increased infectious disease risk and negative values indicating protective effects. (C) Support Vector Machine feature weights, identical to LR due to linear kernel implementation. All three algorithms consistently identified temporal and demographic factors as primary predictors while minimizing the contribution of gender and comorbidity status.

Feature importance evaluation using Random Forest, Logistic Regression, and Support Vector Machine algorithms identified age and visit date as the most influential predictors (Figure 3 and Table 1).

**Table 1:** Feature importance rankings across machine learning algorithms. Values represent the relative importance of four predictor variables (age, gender, visit date, underlying diseases) in Random Forest (RF), Logistic Regression (LR), and Support Vector Machine (SVM) models. Higher absolute values indicate greater predictive importance.

Factors Model			
	RF	LR	SVM
Age	0.3757	−0.652535	−0.652535
Gender	0.0272	−0.069697	−0.069697
Visit date	0.5719	1.079590	1.079590
Underlying diseases	0.025	0.164688	0.164688

Results from the Random Forest model revealed that visit date (importance = 0.5719) and age (importance = 0.3757) were the most important factors, while gender (importance = 0.0272) and underlying diseases (importance = 0.025) showed minimal predictive value (Figure 3A).

The Logistic Regression and Support Vector Machine algorithms produced identical results, showing visit date (coefficient = 1.079590) as the strongest positive predictor and age (coefficient = −0.652535) as having negative importance in predicting infectious disease prevalence before and after the flood (Figures 3B,C). Gender (coefficient = −0.069697) and underlying diseases (coefficient = 0.164688) demonstrated relatively lower importance in these linear models.

The consistent identification of temporal and age factors across different algorithmic approaches supports the robustness of these findings and validates the biological plausibility of the flood‐infection relationship.

### 3.5 Correlation Between Variables

**Figure 4:**
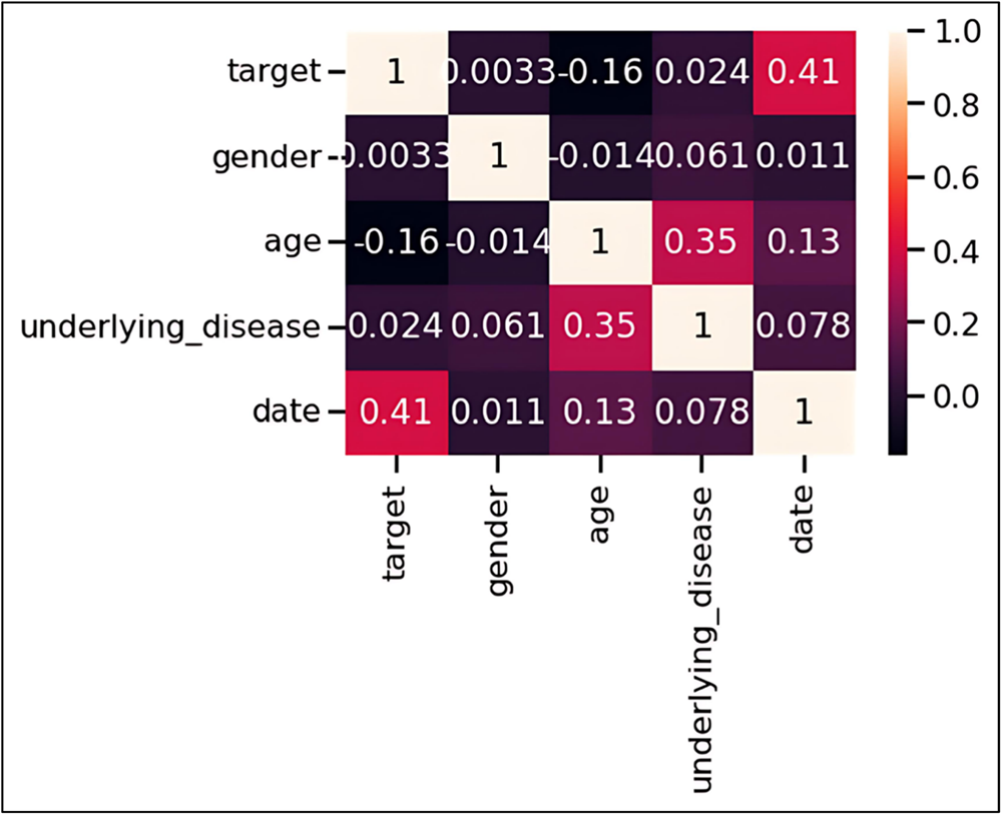
Correlation matrix of study variables using Pearson correlation coefficients. Heat map visualization showing relationships between age, gender, visit date, underlying diseases, and pre‐flood/post‐flood grouping. Color intensity represents correlation strength, with values ranging from −1 (perfect negative correlation) to + 1 (perfect positive correlation). The strongest correlation was observed between age and flood timing (*r* = −0.16), confirming younger patients were more likely to present with infectious diseases in the post‐flood period. Minimal correlations between other variable pairs indicate absence of significant multicollinearity.

Pearson correlation analysis was conducted to examine relationships between study variables (Figure 4). The strongest correlation was observed between age and the pre‐flood/post‐flood grouping variable, with a Pearson correlation coefficient of −0.16. This negative correlation confirms that younger age was associated with the post‐flood period infectious disease cases.

Additionally, age and gender variables showed a weak correlation with a Pearson correlation coefficient of −0.014. The overall correlation structure revealed minimal interdependence among predictor variables, indicating that multicollinearity was not a significant concern in the analysis.

## 4 Discussion

### 4.1 Post‐Flood Infectious Disease Burden and Epidemiological Impact

This study provides quantitative evidence of significant infectious disease burden following flood disasters in Iran. The observed increase from 39.5% to 47.3% infectious disease prevalence represents a meaningful public health impact, with an odds ratio of 1.38 and relative risk of 1.20. The attributable risk of 7.8% points indicates that approximately 1 in 13 post‐flood infectious disease cases could be directly attributed to flood‐related factors.

These findings align with previous systematic reviews documenting increased infectious disease incidence following natural disasters. Saatchi et al. identified similar patterns in their comprehensive scoping review of communicable disease outbreaks after natural disasters, emphasizing the consistent relationship between flooding and infectious disease transmission [2]. Charnley et al. further documented specific traits and risk factors of post‐disaster infectious disease outbreaks in their systematic review [3]. The magnitude of our observed effect (OR = 1.38) falls within the range reported in international literature, though represents a more modest increase compared to some settings, potentially reflecting local healthcare infrastructure capabilities and flood characteristics.

The temporal association observed in our study, with infectious diseases clustering in the immediate post‐flood period, is consistent with established disaster epidemiology patterns. Liu et al. documented similar associations in their worldwide observational study of flood disasters and infectious diseases across 168 countries and territories [1]. However, the specific mechanisms underlying this association require careful interpretation given our study design limitations.

### 4.2 Unexpected Age‐Related Vulnerability Patterns

One of the most striking findings was the counterintuitive age distribution of post‐flood infectious disease cases. Patients in the post‐flood period were significantly younger (mean age 50.99 ± 22.30 years) compared to the pre‐flood period (mean age 57.94 ± 20.25 years), representing a 7‐year difference in mean age. When stratified by age groups, adults aged 18–60 years showed higher post‐flood infectious disease rates compared to those over 60 years. This finding challenges conventional disaster epidemiology assumptions that typically emphasize increased vulnerability among elderly populations (≥ 65 years) [8, 13]. Several potential explanations merit consideration for this unexpected pattern. Younger adults may have experienced greater direct exposure to contaminated floodwater through participation in cleanup activities, property salvage efforts, and disaster response activities. Occupational factors may also contribute, as working‐age individuals often face unavoidable exposure to flood‐affected environments due to employment obligations. Additionally, healthcare‐seeking behaviors may differ across age groups during post‐disaster periods, with younger adults potentially more likely to seek medical attention for infectious symptoms.

This age‐related finding has important implications for disaster preparedness planning. Traditional vulnerability frameworks may need revision to account for exposure‐based risk factors that disproportionately affect working‐age adults. The identification of younger adults as a high‐risk group suggests that public health interventions should include targeted protective measures for individuals engaged in flood cleanup and recovery activities.

### 4.3 Machine Learning Algorithm Performance and Predictive Capability

The comparative evaluation of five machine learning algorithms revealed moderate predictive performance, with the Random Forest model achieving the highest AUC of 0.76. This superior performance of ensemble methods is consistent with recent literature demonstrating their effectiveness in health prediction tasks [4, 5, 7]. Yang et al. reported similar patterns in influenza prediction using multisource data, where ensemble methods outperformed traditional statistical approaches [5]. However, the modest AUC values obtained (0.69–0.76) indicate important limitations in clinical applicability. While these results demonstrate statistically significant discrimination above chance levels, they fall short of the excellent discrimination typically required for clinical decision‐making tools. Al Meslamani et al. noted similar performance limitations in their review of machine learning applications in infectious diseases, emphasizing the complexity of factors influencing disease prediction [7].

The identification of age and visit date as the most important predictive features across algorithms provides insights into the temporal and demographic dimensions of post‐flood infectious disease risk. The consistency of these findings across different algorithmic approaches (Random Forest, Logistic Regression, and Support Vector Machine) supports the robustness of these associations and validates the biological plausibility of the flood‐infection relationship.

### 4.4 Study Limitations and Design Considerations

Several important limitations must be acknowledged. The ecological study design with temporal comparison, while useful for examining population‐level patterns, cannot establish definitive causal relationships between flood exposure and infectious disease incidence. This limitation is inherent to disaster epidemiology research, where prospective cohort studies with pre‐disaster baseline measurements face substantial practical challenges [2]. The constraint to four readily available predictor variables likely contributed to the modest predictive performance observed. Comprehensive prediction models would benefit from incorporating environmental variables (flood depth, duration, water quality), socioeconomic factors (housing conditions, sanitation access), and pathogen‐specific variables (baseline endemicity, seasonal patterns). Charnley et al. emphasized the importance of such multifactor approaches in their systematic review of post‐disaster infectious disease outbreaks [3]. The single‐region design limits generalizability to other flood‐affected populations with different demographic, environmental, or healthcare characteristics. Shokri et al. highlighted the diversity of flood impacts on health across different Iranian populations, suggesting the need for multi‐regional validation studies [10]. The temporal scope of our analysis (30‐day post‐flood period) captures acute infectious disease manifestations but may miss delayed complications or secondary outbreaks. Some infectious diseases have longer incubation periods and may manifest weeks to months after flood exposure [3, 8]. Additionally, our analysis treated infectious diseases as a single aggregate category rather than examining disease‐specific patterns, which would provide more clinically actionable insights. Healthcare utilization data may introduce selection bias, as not all individuals with infectious diseases seek formal healthcare, particularly in disaster‐affected communities [13]. This limitation could affect the representativeness of our findings and may contribute to the observed age patterns if healthcare‐seeking behavior varies across age groups. While these limitations constrain the immediate clinical applicability of our findings, they also highlight important opportunities for advancing disaster epidemiology through improved methodological approaches. The lessons learned from this initial application of machine learning to post‐flood disease prediction inform both immediate public health practice and future research priorities.

### 4.5 Implications for Public Health Practice and Disaster Response

Despite performance limitations, our findings offer actionable insights for disaster preparedness and response planning. The identification of age‐related risk patterns suggests that public health responses should include protective measures specifically designed for working‐age adults engaged in flood cleanup activities. This represents a departure from traditional approaches that primarily focus on elderly and pediatric populations. The temporal clustering of infectious disease cases validates the importance of enhanced surveillance during the immediate post‐flood period. Li et al. emphasized the transformative potential of artificial intelligence in infectious disease management, though noted the need for comprehensive approaches [11]. The predictive models developed in this study, while modest in performance, demonstrate the feasibility of AI‐based approaches in disaster epidemiology. Building on these practical implications, several critical research priorities emerge that could enhance the predictive capability and operational utility of AI‐based disaster epidemiology systems.

### 4.6 Future Research Directions

Several research priorities emerge from this study. Multi‐site validation studies across diverse geographic and climatic regions are needed to assess model generalizability and identify region‐specific risk factors. Integration of comprehensive environmental data, including satellite‐based flood mapping and water quality measurements, could significantly improve predictive accuracy. Disease‐specific modeling approaches would provide more clinically useful predictions for major post‐flood pathogens, including diarrheal diseases, vector‐borne diseases, and respiratory infections. Santangelo et al. highlighted the importance of such targeted approaches in their systematic review of machine learning applications for infectious disease prediction [14]. Future studies should also explore longer follow‐up periods to capture delayed infectious disease complications and examine the potential for real‐time implementation of prediction models in operational disaster response contexts. Economic evaluation of AI‐based prediction systems compared to traditional surveillance approaches would inform resource allocation decisions in disaster preparedness planning.

## 5 Conclusion

This study provides the first comprehensive machine learning analysis of infectious disease prediction following flood disasters in Iran, revealing significant epidemiological burden and challenging conventional vulnerability assumptions. The 20% relative increase in infectious disease risk, coupled with unexpected age‐related patterns favoring younger adults, has important implications for disaster preparedness and response planning. While our machine learning models achieved moderate predictive performance (AUC 0.69–0.76) with Random Forest demonstrating superior discrimination, the modest accuracy levels underscore the complexity of disaster epidemiology and highlight the critical need for more comprehensive prediction systems. The identification of age and healthcare utilization timing as key predictive features provides actionable insights for surveillance system design and resource allocation. The counterintuitive finding of increased infectious disease risk among younger adults challenges traditional disaster vulnerability frameworks and suggests that exposure‐based risk factors may be more important than age‐based susceptibility in flood‐associated infectious disease patterns. This has profound implications for public health intervention strategies, which should prioritize protective measures for working‐age adults engaged in flood response and recovery activities. Future research should focus on developing more comprehensive prediction models incorporating environmental, socioeconomic, and pathogen‐specific variables, while validating approaches across diverse geographic and demographic contexts. The ultimate goal remains the development of clinically actionable prediction systems that can enhance disaster preparedness and reduce the infectious disease burden of flood disasters. Despite current limitations, our findings demonstrate the feasibility and promise of artificial intelligence approaches in disaster epidemiology, providing a foundation for more sophisticated prediction systems that could transform reactive disaster response into proactive public health protection.

## Author Contributions

**Mehdi Safari:** conceptualization, investigation, methodology, formal analysis, writing – review and editing. **Alireza Zali:** conceptualization, writing – review and editing, methodology; investigation, formal analysis. **Hossein Hatami:** writing – review and editing, conceptualization. **Elnaz Amanzadeh Jajin:** data curation, formal analysis, software, writing – original draft. **Meisam Akhlaghdoust:** conceptualization, data curation, investigation, formal analysis, methodology, writing – original draft.

## Conflicts of Interest

The authors declare no conflicts of interest.

## Transparency Statement

The lead author Meisam Akhlaghdoust affirms that this manuscript is an honest, accurate, and transparent account of the study being reported; that no important aspects of the study have been omitted; and that any discrepancies from the study as planned (and, if relevant, registered) have been explained.

## Data Availability

The data that support the findings of this study are available from the corresponding author upon reasonable request.
